# Discovery of small molecule candidate therapeutics against neuroimmune targets in Alzheimer’s disease

**DOI:** 10.1002/alz70859_096004

**Published:** 2025-12-23

**Authors:** Linda Jo Van Eldik, Saktimayee M Roy, Ottavio Arancio, D. Martin Watterson

**Affiliations:** ^1^ University of Kentucky, Lexington, KY USA; ^2^ Northwestern University, Chicago, IL USA; ^3^ Columbia University, New York, NY USA

## Abstract

**Background:**

Dysregulated brain inflammation is a critical mechanism driving neurodegenerative disease progression. Neuroinflammatory processes are fundamental to CNS homeostasis, but inflammation that is inefficient, excessive, or prolonged is detrimental. Stressor‐induced proinflammatory cytokine overproduction is an explicit form of neuroinflammation linked to activated glia and synaptic dysfunction in Alzheimer’s disease (AD). We have explored pharmacological mechanisms that could attenuate dysregulated proinflammatory cytokine production and restore glia‐neuronal homeostasis. We used both unbiased phenotypic and single molecular target approaches to discover and develop unique therapeutic candidates that selectively modulate panels of proinflammatory cytokines linked to pathophysiology susceptibility or progression.

**Method:**

We designed and synthesized strategic collections of unique small molecules based on the privileged pyridazine chemotype and pharmacoinformatics. Recursive screens and medicinal chemistry refinements were driven by secondary pharmacology assays integrated with in vivo efficacy and pharmacodynamics measurements in diverse animal models. The independent parallel approaches delivered a series of candidates for disease‐focused development. All deliverables exhibited oral bioavailability and brain exposure, promising safety profiles, and efficacy at low doses in animal models of CNS disorders.

**Result:**

Three therapeutic candidates, MW150, MW151, and MW189, are in human phase 1b/2a clinical trials.

**Conclusion:**

Our drug discovery efforts have taught us several important lessons:

1) Focus efforts on disease‐relevant pathophysiology processes integrated with strategic compound design.

2) Fail fast and early.

3) Complex diseases such as AD require interventions that target complementary disease drivers and therapeutic intervention time windows.

4) Dosing is the pharmacological basis of successful therapeutic intervention: what, when, and how – the ***RIGHT*** drug(s) at the ***RIGHT*** time(s) for the ***RIGHT*** disease mechanism.

5) The cytokine/synaptic dysfunction axis is a viable pathophysiology process for therapeutic intervention where proinflammatory cytokine dysregulation is part of the disease progression mechanism.

